# Initial Clinical Experience With AneuFix Injectable Biocompatible Elastomer for Translumbar Embolization of Type 2 Endoleaks

**DOI:** 10.1177/15266028231165731

**Published:** 2023-04-19

**Authors:** Stefan P. M. Smorenburg, Rutger J. Lely, Bas-Jeroen van Kelckhoven, Erik G. Vermeulen, Kak Khee Yeung, Rombout R. Kruse, Martin Kraai, Chrit M. Stassen, Michael J. Jacobs, Arjan W. J. Hoksbergen

**Affiliations:** 1 Department of Surgery, Amsterdam University Medical Centers location, Vrije Universiteit Amsterdam, Amsterdam, the Netherlands; 2 Amsterdam Cardiovascular Sciences, Amsterdam, the Netherlands; 3 Department of Radiology, Amsterdam University Medical Centers location Vrije Universiteit Amsterdam, Amsterdam, the Netherlands; 4 Department of Radiology, Spaarne Gasthuis Hospital, Haarlem, the Netherlands; 5 Department of Surgery, Spaarne Gasthuis Hospital, Haarlem, the Netherlands; 6 Department of Surgery, Hospital Group Twente, Almelo-Hengelo, the Netherlands; 7 Department of Radiology, Hospital Group Twente, Almelo-Hengelo, the Netherlands; 8 Department of Vascular Surgery, Maastricht University Medical Center+, Maastricht, the Netherlands; 9 Department of Vascular Surgery, European Vascular Center Aachen-Maastricht, University Hospital RWTH Aachen, Aachen, Germany

**Keywords:** elastomer, polymer, embolization, type II endoleak, translumbar, abdominal aortic aneurysm, endovascular aneurysm repair, abdominal aortic aneurysm, AAA growth

## Abstract

**Purpose:**

The aim of this study was to assess the initial experience, technical success, and clinical benefit of AneuFix (TripleMed, Geleen, the Netherlands), a novel biocompatible and non-inflammatory elastomer that is directly injected into the aneurysm sac by a translumbar puncture in patients with a type II endoleak and a growing aneurysm.

**Materials and Methods:**

A multicenter, prospective, pivotal study was conducted (ClinicalTrials.gov:NCT02487290). Patients with a type II endoleak and aneurysm growth (>5 mm) were included. Patients with a patent inferior mesenteric artery connected to the endoleak were excluded for initial safety reasons. The endoleak cavity was translumbar punctured with cone-beam computed tomography (CT) and software guidance. Angiography of the endoleak was performed, all lumbar arteries connected to the endoleak were visualized, and AneuFix elastomer was injected into the endoleak cavity and short segment of the lumbar arteries. The primary endpoint was technical success, defined as successful filling of the endoleak cavity with computed tomography angiography (CTA) assessment within 24 hours. Secondary endpoints were clinical success defined as the absence of abdominal aortic aneurysm (AAA) growth at 6 months on CTA, serious adverse events, re-interventions, and neurological abnormalities. Computed tomography angiography follow-up was performed at 1 day and at 3, 6, and 12 months. This analysis reports the initial experience of the first 10 patients treated with AneuFix.

**Results:**

Seven men and 3 women with a median age of 78 years (interquartile range (IQR), 74-84) were treated. Median aneurysm growth after endovascular aneurysm repair (EVAR) was 19 mm (IQR, 8–23 mm). Technical success was 100%; it was possible to puncture the endoleak cavity of all treated patients and to inject AneuFix. Clinical success at 6 months was 90%. One patient showed 5 mm growth with persisting endoleak, probably due to insufficient endoleak filling. No serious adverse events related to the procedure or AneuFix material were reported. No neurological disorders were reported.

**Conclusion:**

The first results of type II endoleak treatment with AneuFix injectable elastomer in a small number of patients with a growing aneurysm show that it is technically feasible, safe, and clinically effective at 6 months.

**Clinical Impact:**

Effective and durable embolization of type II endoleaks causing abdominal aortic aneurysms (AAA) growth after EVAR is challenging. A novel injectable elastic polymer (elastomer) was developed, specifically designed to treat type II endoleaks (AneuFix, TripleMed, Geleen, the Netherlands). Embolization of the type II endoleak was performed by translumbar puncture. The viscosity changes from paste-like during injection, into an elastic implant after curing. The initial experience of this multicentre prospective pivotal trial demonstrated that the procedure is feasible and safe with a technical success of 100%. Absence of AAA growth was observed in 9 out of 10 treated patients at 6 months.

## Purpose

Type II endoleaks (T2ELs) are defined as retrograde flow from aortic side-branch vessels into the native abdominal aortic aneurysm (AAA) sac after endovascular aneurysm repair (EVAR).^1^ T2ELs are the most prevalent complications occurring in 16% to 50% patients after EVAR.^2–4^ A large proportion of T2ELs resolves spontaneously over time up to 58%.^2^ However, another portion remains at risk for persistent aneurysm growth potentially compromising proximal or distal seal zones.^5^ The risk of late rupture caused by T2ELs that do not progress to type I or III endoleaks remains low at 0.9% (95% confidence interval [CI], 0.77 to 1.05).^6,7^

The current guidelines by the Society for Vascular Surgery advocate only intervention for patients with persistent T2ELs and a sac growth of >5 mm.^8^ The European Society for Vascular Surgery recommends a more conservative approach with only intervention after >10 mm sac growth.^4^ When diagnosed, lifelong yearly imaging is advised to monitor growth.^4,8^

Type II endoleak treatment options are transarterial or transcaval embolization, translumbar embolization by direct percutaneous puncturing of the aneurysm(al) sac, laparoscopic clipping, or open ligation after saccotomy.^5,9,10^ Several different embolization materials are available and used, such as glue, coils, thrombin, gelfoam, and Onyx (Medtronic, Minneapolis, USA).^11–17^ Two recent meta-analyses reported a relatively high technical success for these procedures (87.4%–87.9%); however, the clinical success defined as aneurysm stabilization or shrinkage was significantly lower (53.5%–68.4%).^18–20^ The embolic materials used in these studies were initially not developed for aneurysm sac embolization and were used off-label.

The aim of this study was to report the initial experience and short-term outcomes of AneuFix (TripleMed, Geleen, the Netherlands), a novel biocompatible and non-inflammatory elastomer that is specifically developed for injection into the aneurysm sac by a translumbar puncture in patients with a T2EL and persistent aneurysm growth after EVAR.

## Materials and Methods

### Study Design and Patients

The AneuFix study is an ongoing multicenter, prospective, pivotal study initiated at Amsterdam University Medical Centres location VUmc, Spaarne Gasthuis Hospital Haarlem and Hospital Group Twente Almelo, the Netherlands.

The study includes patients treated with EVAR and persistent growth of the aneurysm sac based on a lumbar T2EL. Patients with a patent inferior mesenteric artery (IMA) connected to the endoleak were excluded. Therapeutic consensus for treatment of type 2 endoleak with an open IMA is to coil the IMA and observe its effect. Together with the potential risk of unintended IMA embolization, this was the main reason to exclude patients with an open IMA connected to the endoleak. All patients were informed on the procedure details, and written consent was obtained after shared decision making. A detailed list of the eligibility criteria is given in Supplementary Table S1. The institutional review board approved the trial protocol, and the study was performed in accordance with the Declaration of Helsinki. The trial has been registered at ClinicalTrials.gov with identifier NCT02487290 and approved by the Dutch Central Committee on Research Involving Human Subjects in accordance with the recent implemented European Union Medical Device Regulation. We report our initial experience of the first 10 consecutive patients treated with AneuFix during this trial.

### AneuFix Biocompatible Elastomer With Changing Viscosity

AneuFix is a 2-component biocompatible and non-inflammatory elastic polymer. The material is based on polydimethylsiloxane (PDMS), which is widely applied for long-term implants because of its biological inertness, stability, and availability in several viscosities.^21,22^ It has the unique properties to change viscosity during the translumbar embolization procedure, from low viscosity (paste like) during injection to high viscosity after injection and after 2 minutes cures to a solid, flexible, and compliant implant. AneuFix has been extensively studied in preclinical tests and demonstrated to be biocompatible, biostable, and has a thrombogenicity comparable to expanded polytetrafluorethylene (ePTFE); a material widely used in current aortic stent grafts.^22,23^ Before clinical application, to prove feasibility, several in vitro and in vivo experiments with AneuFix have been conducted and published.^22–27^ The material was specifically developed for injection in the circulated aneurysm sac, where it fills the cavity of the endoleak and ostia of lumbar arteries with high controllability.

AneuFix is an implant intended for long-term use and is in direct contact with the circulatory system. As such, the implant was classified as a class III medical device per Rule 8 of Annex IX to Medical Device Directive 93/42/EEC as amended by 2007/47/EC. In the European Union, these medical devices require CE certification by the notified body before introduction into the market. As this is a pivotal trial, AneuFix is pre-CE mark.

**Figure 1.**
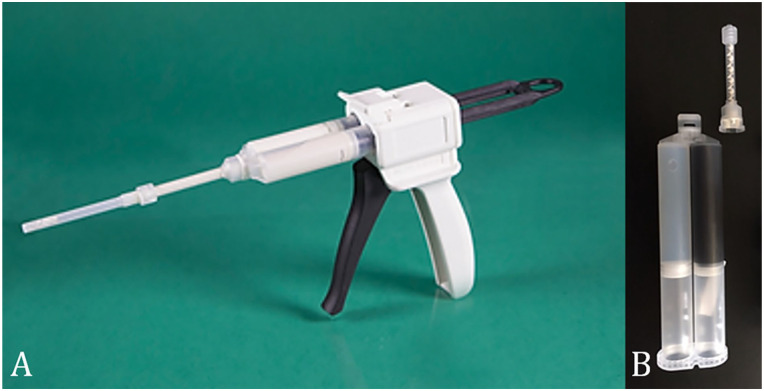
Dispenser for injection (A) and syringe with AneuFix 2-component polymer (B).

The AneuFix-kit contains a double-barrel 2 × 20 mL syringe with the elastomer, a dispenser gun, and a static mixer which is mounted onto the dispenser (Figure 1A and B). During injection, both components are mixed in the static mixer through a plastic helix, after which the curing process starts. The viscosity of each of the 2 elastomer components is high at rest, but when mixed together and injected under pressure, this material can be injected through a needle typically used during translumbar interventions such as an Access Trocar Needle 18G (Mermaid Medical, Stenløse, Denmark). During injection with AneuFix, the polymer has gel-like properties. When it enters the lumbar artery, injection can be paused to let the material solidify, after which injection can be resumed. Immediately after injection, the material starts recovering to its higher viscosity state. AneuFix contains 30% tantalum, which is an inert metal, to ensure real-time visualization under fluoroscopic guidance.

### Translumbar Embolization and Aneurysm Sac Angiography

**Figure 2.**
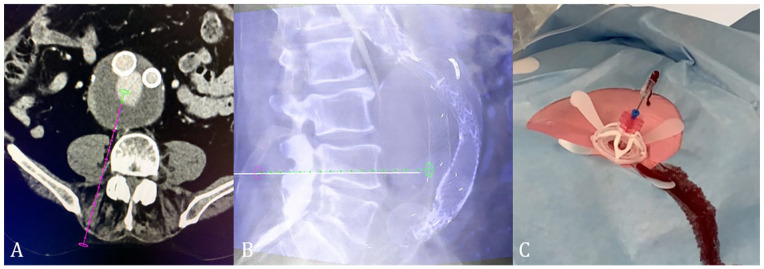
The needle puncture route (A) on preoperative computed tomography angiography (CTA), with needle guidance software based on intraoperative cone-beam computed tomography (CT) (B), and return of blood from the needle, indicating puncture of endoleak cavity (C).

The percutaneous translumbar puncture route was determined on preoperative multiphase abdominal computed tomography (CT). The arterial and delayed-phase series were used to visualize the endoleak with connecting lumbar arteries. Depending on the puncture route (Figure 2A), patients were positioned prone, or on the right or left side. The first 2 patients were treated under general anesthesia, while all other patients under local anesthesia. A contrast-enhanced or blank cone-beam CT to locate the endoleak was performed in the hybrid operating room. The scans were obtained at 35 and 70 seconds after intravenous contrast injection. The contrast-enhanced cone-beam CT, or fusion between blank cone-beam CT and preoperative computed tomography angiography (CTA), was utilized to locate the endoleak cavity and corresponding lumbar arteries. Then, dedicated needle guidance software was used to define a target location and entry point (Figure 2B), XperGuide (Philips, Best, the Netherlands) and Syngo Needle Guidance (Siemens, München, Germany).

In this trial, it was the choice of the treating physician if contrast enhanced, or a blank cone-beam computed tomography (CBCT) was used for needle guidance. Fusion of the CBCT with preoperative CTA was also possible and occasionally used. The needle route was checked in axial, lateral, and frontal planes to avoid puncture of unwanted anatomical structures. After puncturing the endoleak cavity, the needle outflow was checked on the presence and pulsatile return of blood (Figure 2C).

**Figure 3.**
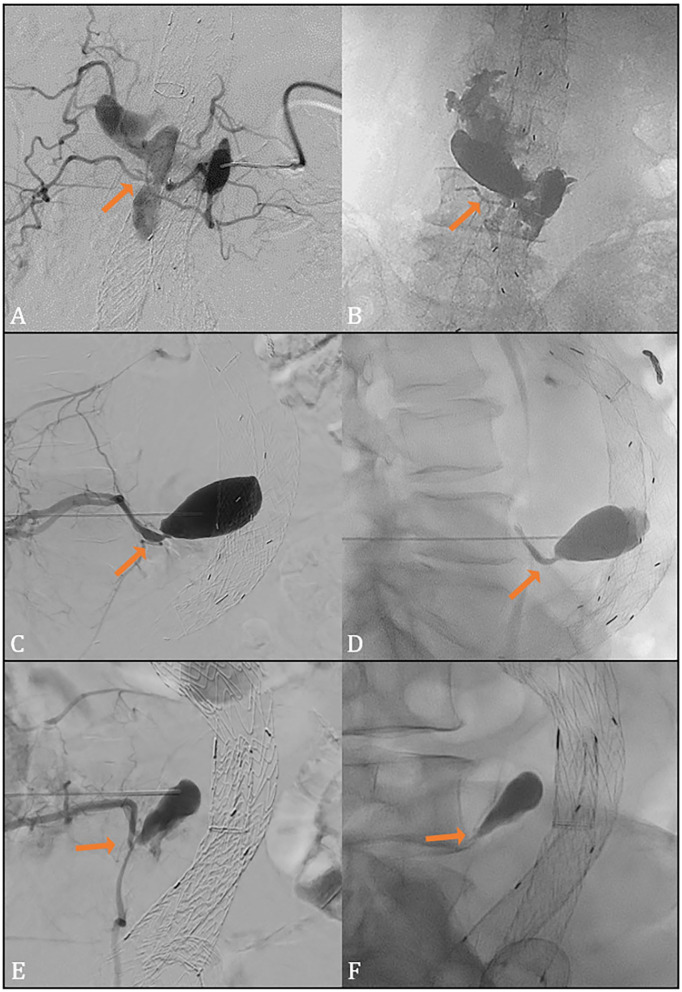
Angiogram of the endoleak cavity (A, C, E) with connecting lumbar arteries (arrow) and fluoroscopic result after AneuFix injection (B, D, F) with filling of small section of a feeding lumbar artery (arrow). Directions of view are anterior-posterior (A, B) and lateral (C–F).

After this, an angiogram of the endoleak cavity within the aneurysm sac was performed (saccogram) by injecting 10 mL Ultravist contrast (300 mg I/mL, UltraVist, Bayer HealthCare AG, Berlin, Germany) (Figure 3A, C, and E). The lumbar arteries were localized, and the shape of the endoleak cavity was outlined manually or digitally.

The static mixer tip was connected to the connecting top of the syringe, to assure that both polymer materials were mixed appropriately and to guarantee a standardized curing process, after which the double syringe was placed into the dispenser.

Under fluoroscopic guidance, the AneuFix material was injected into the endoleak cavity with a steady continuous flow. During injection, the AneuFix mass increases in size. Injection was temporarily stopped when AneuFix passed into a small segment of the lumbar arteries, to enhance local curing and prevent distal embolization. The risk of distal non-target embolization through a lumbar artery is spinal cord ischemia with paraplegia, which is why the lumbar arteries function as guiding and reference point during injection, and injection is paused when the material entered the lumbar artery ensuring that only a short segment was embolized. After this, injection was continued to match the outline shape of the saccogram, as optimally as possible. When the endoleak outline of the initial angiogram was reached, the injection was ended (Figure 3B, D, and F). Finally, the needle was retracted with vacuum under pressure. To visualize the completed shape of the polymer, a cone-beam CT (blank) was performed. All procedures were proctored by AH, RL, SS and a representative of TripleMed. The cured AneuFix implant has a highly compliant elasticity and in long time follow-up in previously published animal studies, retained its original elasticity without any sign of microfractures or loss of surface integrity.^23–27^

### Primary Endpoint

Primary endpoint of the study was technical success, defined as successful filling of the endoleak cavity with polymer. This was assessed by means of a CT scan within 24 hours after the completion of the injection procedure.

### Secondary Endpoints

The main secondary endpoint was clinical success, which was defined as the absence of aneurysm sac growth (>5 mm), measured as maximum aneurysm diameter (orthogonal on multiplanar reconstruction) at 6 months on CTA. All measurements were performed by an independent imaging core lab. Further secondary endpoints were as follows: documentation of intra- and peri-operative complications (<30 days); occurrence of serious adverse events; vascular-related adverse events; adverse device effects; complications and deaths; neurological complaints; re-interventions; aneurysm rupture in the peri-operative period; occurrence of general adverse events and adverse device effects at 1, 3, 6, 12, and 24 months; rate of secondary endovascular or surgical re-interventions at 1, 3, 6, and 12 months; and survival throughout the study up until 24 months. A detailed list of adverse events definitions is given in Supplementary Table S2.

### Measurements and Follow-up

#### Screening assessments

**Figure A1.**
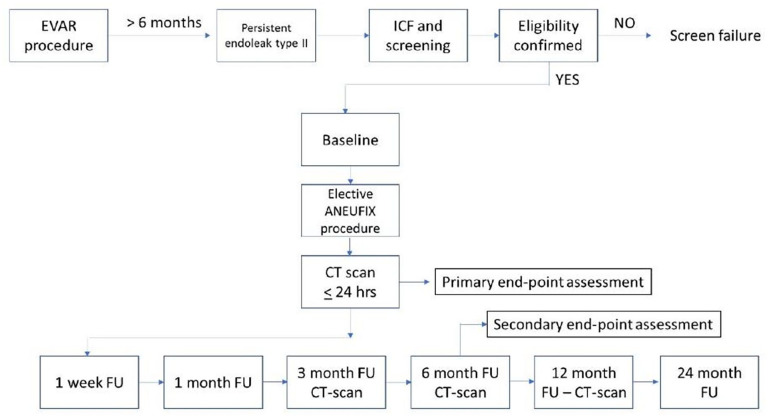
Flowchart of study design.

After informed consent, patient screening was performed, and the following information was collected: demographic data, relevant medical history, comorbidities, relevant current medication, blood lab estimated glomerular filtration rate (eGFR), and serum creatinine. Also, the AAA diameter was determined from the first postoperative CTA post-EVAR to determine pre-AneuFix AAA growth. The AneuFix injection procedure was performed within 3 months of screening assessments. A flowchart of the study is displayed in Figure A1 in Appendix A.

#### Baseline assessments

The baseline assessments were performed on the day of the AneuFix injection, prior to the procedure. Typically, the patients were hospitalized early in the morning on the day of the procedure. Baseline assessments consisted of a neurologic examination: functioning, sensitivity, and mobility of lower back/upper legs conducted by an independent physician, blood lab C-reactive protein (CRP), creatine kinase (CK), and adverse events since screening visit. A detailed list of all investigation procedures is given in Supplementary Table S3.

#### Procedural assessments

The local practice with respect to withholding/administration of anti-thrombolytic medication during the procedure was considered when a patient was on a regime of oral anti-coagulant medication prior to the procedure. During the procedure, the following assessments were gathered: patient position, needle distance, blood outflow, AneuFix injection time, AneuFix volume, lumbar artery involvement, needle length, inner diameter, fluoroscopy time, radiation exposure, contrast volume, and procedure time.

#### Post-procedure until hospital discharge (<24 hours after procedure)

The patient was kept overnight in order to perform a CTA the next morning. The follow-up CTA settings were thin-sliced (0.625–1.0 mm) with a blank, arterial and venous phase, with an artifact suppression protocol and 70 mL iodine-based contrast (Xenetix 350 mgI/mL, Guerbet, Villepinte, France) and the AneuFix position, endoleak presence and type was scored. Furthermore, the neurological examination was repeated and compared with preprocedural assessment (functioning, sensitivity, mobility of lower back/upper limbs), blood lab CRP, and CK eventual and adverse events were evaluated.

#### Follow-up 1 week and 1 month

At 1 week, the CRP was assessed, along with relevant medication changes and adverse events. At 1 month, similar assessments were performed except for the CRP.

#### Follow-up 3 months with CTA

At 3 months, CTA was repeated and aneurysm diameter, and endoleak presence and type were assessed.

#### Follow-up 6 months with CTA (clinical success)

Computed tomography angiography at 6 months was performed to assess aneurysm diameter, endoleak presence and type, and clinical success. Sac stabilization was defined as growth <5 mm throughout the follow-up period. Potential adverse events were also reported.

#### Follow-up 12 months with CTA

The final CTA was performed at 12 months. Aneurysm diameter was measured, and endoleak presence and type and adverse events were reported.

#### Follow-up: 24 Months

Following 2 years after the AneuFix injection, adverse events will be assessed that may have occurred since the previous follow-up (FU) visit such as hospital re-admission and to assess survival.

Regular ultrasound or CTA follow-up should be performed, based on the guidelines and physicians’ or hospitals’ preference.

### Statistical Methods

Normal distributed continuous outcome variables were summarized by their mean and standard deviations, and categorical variables were summarized as proportions. Not normal distributed continuous outcome variables were summarized by the median and interquartile range (IQR) with its first and third percentile or range.

The outcome of all treated patients was represented as a proportion of technical and clinical success.

## Results

**Figure 4.**
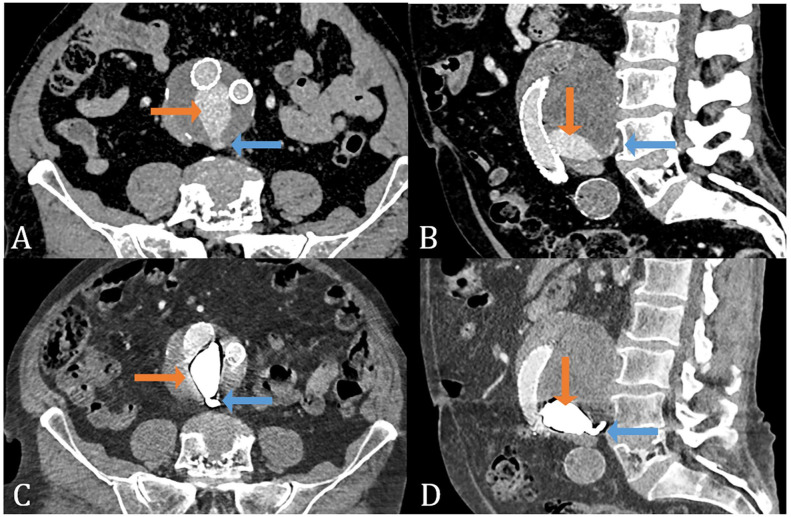
Preoperative computed tomography angiography (CTA) with type II endoleak (A,B) and connecting lumbar arteries (blue arrow) with endoleak cavity (orange arrow) and postoperative CTA with AneuFix treatment (C,D) and treated lumbar arteries (blue arrow) with treated endoleak cavity (orange arrow).

Between March 2020 and April 2021, the first 10 patients were treated with AneuFix. The majority were men (n=7) with a median age of 78 years (range, 71–90), and all baseline characteristics are provided in Table 1. The CTA result after AneuFix embolization is displayed in axial (Figure 4C) and sagittal views (Figure 4D), with corresponding before AneuFix CTA images in Figure 4A and B.

**Table 1.** Patient Demographics and Comorbidities.

Characteristic	Cases (n=10)	
Age, years	77.5	(74–84)
Man	7	(70)
Woman	3	(30)
Cardiovascular history		
Hypertension	7	(70)
Coronary artery disease	4	(40)
Myocardial ischemia	3	(30)
Atrial fibrillation	2	(20)
Hypercholesterolemia	3	(30)
Peripheral artery disease	1	(10)
General history		
Carcinoma	4	(40)
Chronic obstructive pulmonary disease	4	(40)
Diabetes	5	(50)
Kidney disease	2	(20)
Baseline eGFR (CKD-EPI), mL/min	72.5	(58–88)

Categorical variables are presented as number (%). Continuous variables are presented as median (interquartile range).

Abbreviations: CKD-EPI, Chronic Kidney Disease Epidemiology Collaboration; eGFR, estimated glomerular filtration rate.

All T2ELs originated from the lumbar arteries. Before the AneuFix procedure, several interventions had been performed to treat various endoleaks (Table 2). In 4 patients, the IMA was embellished with coils. One patient underwent 3 lumbar artery embolization procedures, without success. Two patients had a type 1b endoleak and had been treated with stent graft leg extension. Two patients had a type 1a endoleak, of which one was treated with a fenestrated proximal extension, and one patient with a proximal cuff. After these interventions, aneurysm growth remained because of an isolated T2EL; hence, these patients were included in the study and treated with AneuFix. The median aneurysm sac size increase after EVAR was 19 mm (IQR, 8–23 mm), and the median number of patent lumbar arteries was 5.5 (IQR, 4.3–6.0).

**Table 2.** Type II Endoleak Origin and Endovascular History.

Patient	Endoleak type II origin	Amount of patent lumbar arteries	AAA size post-EVAR (mm)	AAA size pre-AneuFix (mm)	Endovascular interventions post-EVAR, pre-AneuFix
1	Lumbar+IMA (coiled)	8	67	90	Embolization IMA
2	Lumbar	8	67	81	Coiling lumbar arteries (first attempt, failed) Coiling lumbar arteries (second attempt, one artery) FEVAR stent above EVAR (type 1A endoleak) Coiling lumbar arteries (third attempt, partial success) Extending right EVAR leg (type 1B endoleak)
3	Lumbar+IMA (coiled)	5	92	98	Proximal cuff with endoanchors (type 1 endoleak) Embolization IMA Extending right EVAR leg (type 1B endoleak)
4	Lumbar	4	64	85	—
5	Lumbar	5	60	63	—
6	Lumbar	6	63	71	—
7	Lumbar	4	57	76	—
8	Lumbar	6	58	67	—
9	Lumbar+IMA (coiled)	6	55	84	Embolization IMA+histoacryl in aneurysm sac
10	Lumbar+IMA (coiled)	4	59	78	Embolization IMA

Abbreviations: AAA, abdominal aortic aneurysm; (F)EVAR, (fenestrated) endovascular aneurysm repair; IMA, inferior mesenteric artery.

During the AneuFix procedure, 4 patients were positioned prone, 4 on the right side, and 2 on the left side. All procedural details are provided in Table 3. The first 2 patients were treated under general anesthesia, and the other 8 under local anesthesia. After translumbar puncture, blood pulsatility from the needle was present in 8 patients. The median AneuFix volume injected was 11 mL (range, 6–20 mL), and median AneuFix injection time was 6 minutes (range, 3–18 minutes). During the injection, fluoroscopy was continually utilized with a total median fluoroscopy time of 8 minutes (range, 3–20 minutes) and median radiation exposure of 395 mGy (range, 253–1404 mGy). The total median procedure time was 1 hour and 27 minutes (range, 45 minutes–2 hours 20 minutes) and in total, a median of 100 mL contrast volume was used (range, 5–120 mL), depending on the utilization of a contrast-enhanced cone-beam CT or cone-beam CT without contrast. No neurological abnormalities were observed pre or post AneuFix, assessed by an independent physician according to a standardized protocol.

**Table 3.** Procedure Details and Technical and Clinical Success.

Procedure information	Cases (n=10)	
Patient position		
Prone	4	
Right side	4	
Left side	2	
Anesthesia		
General	2	
Local	8	
Translumbar puncture		
Pulsatile blood from needle	8	
No pulsatile blood from needle	2	
Needle length 15 cm	5	
Needle length 20 cm	5	
AneuFix volume injected, mL	11	(6–20)
Duration of AneuFix injection, min	6	(3–18)
Imaging information		
Fluoroscopy time, min	8	(3–20)
Radiation exposure (air kerma), mGy	395	(253–1404)
General		
Neurological abnormalities	0	
Contrast volume, mL	100	(5–120)
Procedure time (entry, exit), h:mm	1:27	(0:45–2:20)
Residual endoleak after procedure	7/10	
Technical success^a^	10/10	
Clinical success^b^	9/10	

Categorical variables are presented as number. Continuous variables are presented as median (range).

Abbreviations: CTA, computed tomography angiography.

a Successful filling of the endoleak cavity with polymer. This was assessed by means of a CTA scan within 24 hours after the completion of the injection procedure.

b Absence of aneurysm sac growth at 6-month CTA based on independent imaging core lab assessments, measured as maximum aneurysm diameter (double oblique, multiplanar reconstruction).

### Primary Endpoint

Technical success was achieved for all 10 patients (100%); it was possible to puncture the intended primary endoleak cavity of all patients and to fill the endoleak with AneuFix (Table 3).

### Secondary Endpoints

At 6-month follow-up (clinical success), there was absence of aneurysm growth in 9 of the 10 patients (Table 3). During follow-up, one patient showed 5 mm growth and a persistent T2EL on CTA probably due to incomplete polymer filling. Another six patients demonstrated a small residual or new endoleak without aneurysm growth.

Furthermore, no related serious adverse events, neurological complaints, or ruptures were reported. One patient with multiple comorbidities died of myocardial infarction and urosepsis 12 months after treatment, which was not related to the procedure. Reported adverse events were back pain after the procedure related to the needle puncture in one patient which resolved over time. Furthermore, one patient had abdominal pain and one patient had a urinary tract infection which also resolved over time and with antibiotics, respectively.

## Discussion

Our initial experience with AneuFix to treat T2ELs in patients with persistent aneurysm growth after EVAR showed that the procedure was feasible, safe, and clinically effective at 6 months in the majority of the patients. To date, numerous treatment options are available for patients with T2ELs. The overall technical success is high (87.9%), but the clinical success remains moderate (68.4%) as demonstrated by a meta-analysis of Ultee et al^19^ in 1073 patients. When stratified for method of treatment, translumbar endoleak treatment is superior over transarterial embolization, which was demonstrated by a recent meta-analysis of Guo et al in 354 patients with 100% technical success for translumbar embolization versus 74.7% technical success for transarterial embolization. Furthermore, clinical success of translumbar versus transarterial embolization was also higher (66% vs 44%, respectively) but not significant.^18^ In a recent study of Charitable et al, a case-series was published of solely translumbar T2EL treatment with cyanoacrylate glue and coils showing resolution of the endoleak and sac stabilization in 50% (n=30) and persistent T2ELs with sac stabilization in 36.7% at 21 months. Another recent case-series published by Fanelli et al^28^ demonstrated sac shrinkage at 1 year in 68% and sac stabilization in 32% in 50 patients treated with Onyx+microcoils.

In the literature, different definitions of technical and clinical success are used. Most definitions focus on successful embolization of the endoleak nidus, and some definitions include embolization of the inflow/outflow vessels. Clinical success depends on aneurysm growth, stabilization, or regression. Usually, a margin of 5 mm is defined to incorporate the measurement error; however, this is not always mentioned, hampering comparison of the outcomes.

Worldwide, many different embolic materials are used for active sac management, with coils, glue, thrombin, and Onyx being the most predominant for T2ELs.^18,29^ What differentiates AneuFix from the aforementioned materials is the changing of viscosity and material properties during the procedure, from fluid state during injection to a flexible solid state after injection in situ. The advantage of this is more material control during injection, to prevent non-target embolization. Fanelli et al^28^ describe inserting multiple coils in the aneurysm sac in large AAAs to prevent Onyx from embolizing. These actions are not needed with AneuFix, which is specifically developed for use in AAAs without additional coil packing. Technically, it is possible to combine coils with AneuFix; however, the aim of this procedure was to have a simple, 1-step procedure for type 2 endoleak treatment. Most of the current materials are being used off-label to treat endoleaks and were not specifically developed for such a purpose. In addition, casting fractures during follow-up were detected in Onyx after embolization of endoleaks, which might compromise long-term integrity.^30^

During our initial experience with AneuFix, it was possible to puncture the endoleak cavity of all 10 patients resulting in a high technical success (100%). At 6-month CTA, 9 of the 10 patients demonstrated absence of aneurysm growth (>5 mm), revealing a high clinical success (90%). However, results of the effectiveness should be interpreted with caution given the limited study population and follow-up. One patient did not demonstrate aneurysm sac stabilization; we hypothesize that this might have been caused by too conservative AneuFix filling in this patient, possibly related to the learning curve of the procedure. Complete filling of the primary endoleak cavity including a short segment of the feeding lumbar arteries is important for successful closure of the endoleak.

During the procedure, we experienced that we could treat the main endoleak cavity and mostly a short section of the lumbar arteries. However, we found that there were sometimes multiple endoleak locations; a primary large nidus and a very small endoleak located more proximal, not necessarily connected to the primary nidus. During the planning and treatment phase, the primary endoleak nidus was targeted and treated. However, we experienced, during the 6-month follow-up, that there were small residual or new endoleaks in 7 patients of which one showed continuous aneurysm growth (Table 3). The primary endoleak was treated and given the short follow-up period of this study; it is unclear what the clinical consequence of the small endoleaks is. If the aneurysm sac remains stable and does not grow further, it was considered a clinical success.

### Limitations

There are several limitations to this study. First, we report our initial experience in a limited study population. We have demonstrated feasibility and safety, however, results on clinical success, although hopeful, should be interpreted with caution. Second, the translumbar puncture contained some variability as some physicians used contrast-enhanced cone-beam CT for needle guidance, and others used a blank cone-beam CT with preoperative CTA fusion as guidance. Both methods, however, demonstrated that it was possible to puncture the aneurysm sac. Third, there is a learning curve to perform the injection of AneuFix material.

### Future Developments

Given the novelty of the AneuFix material, it remains under continuous development and improvements. First, the amount of tantalum metal will be altered in order to reduce scatter in follow-up CTA. Several studies are currently running to investigate the optimum amount of tantalum. Second, since this report only encompasses the first 10 consecutive patients, a final publication with all patients treated with AneuFix and completed follow-up will be published to share definite technical and longer term clinical success.

## Conclusion

The first results of T2EL treatment with AneuFix injectable biocompatible elastomer in 10 patients with a growing aneurysm show that it is technically feasible, safe, and clinically effective at 6 months.

## Supplemental Material

sj-docx-1-jet-10.1177_15266028231165731 – Supplemental material for Initial Clinical Experience With AneuFix Injectable Biocompatible Elastomer for Translumbar Embolization of Type 2 Endoleaks Supplemental material, sj-docx-1-jet-10.1177_15266028231165731 for Initial Clinical Experience With AneuFix Injectable Biocompatible Elastomer for Translumbar Embolization of Type 2 Endoleaks by Stefan P. M. Smorenburg, Rutger J. Lely, Bas-Jeroen van Kelckhoven, Erik G. Vermeulen, Kak Khee Yeung, Rombout R. Kruse, Martin Kraai, Chrit M. Stassen, Michael J. Jacobs and Arjan W. J. Hoksbergen in Journal of Endovascular Therapy

sj-docx-2-jet-10.1177_15266028231165731 – Supplemental material for Initial Clinical Experience With AneuFix Injectable Biocompatible Elastomer for Translumbar Embolization of Type 2 Endoleaks Supplemental material, sj-docx-2-jet-10.1177_15266028231165731 for Initial Clinical Experience With AneuFix Injectable Biocompatible Elastomer for Translumbar Embolization of Type 2 Endoleaks by Stefan P. M. Smorenburg, Rutger J. Lely, Bas-Jeroen van Kelckhoven, Erik G. Vermeulen, Kak Khee Yeung, Rombout R. Kruse, Martin Kraai, Chrit M. Stassen, Michael J. Jacobs and Arjan W. J. Hoksbergen in Journal of Endovascular Therapy

sj-docx-3-jet-10.1177_15266028231165731 – Supplemental material for Initial Clinical Experience With AneuFix Injectable Biocompatible Elastomer for Translumbar Embolization of Type 2 Endoleaks Supplemental material, sj-docx-3-jet-10.1177_15266028231165731 for Initial Clinical Experience With AneuFix Injectable Biocompatible Elastomer for Translumbar Embolization of Type 2 Endoleaks by Stefan P. M. Smorenburg, Rutger J. Lely, Bas-Jeroen van Kelckhoven, Erik G. Vermeulen, Kak Khee Yeung, Rombout R. Kruse, Martin Kraai, Chrit M. Stassen, Michael J. Jacobs and Arjan W. J. Hoksbergen in Journal of Endovascular Therapy
