# Supplementary material for: Initial Clinical Experience With AneuFix Injectable Biocompatible Elastomer for Translumbar Embolization of Type 2 Endoleaks
Source: J Endovasc Ther. 2023 Apr 19;32(1):57–67. doi: 10.1177/15266028231165731 (PMC11707960; doi:10.1177/15266028231165731)
Supplement: sj-docx-1-jet-10.1177_15266028231165731 – Supplemental material for Initial Clinical Experience With AneuFix Injectable Biocompatible Elastomer for Translumbar Embolization of Type 2 Endoleaks [file sj-docx-1-jet-10.1177_15266028231165731.docx]

## S1. Inclusion/exclusion criteria table.

| **Inclusion criteria:**  Patients are eligible for study participation if they comply with ALL of the inclusion criteria:  1. Persistent type II endoleak (more than 6 months post-EVAR or post-embolization  procedure3); AND  2. Volume of the ‘endoleak cavity’ can be estimated upfront; AND  3. An EVAR without circulatory complications (including the absence of an open AMI that is connected to the endoleak as judged on existing CT scan); AND  4. An endoleak confirmed on a CT scan that should be done within 180 days prior to  procedure but preferably within 90 days prior to screening demonstrating the high  likelihood of the isolated nature of the endoleak; AND  5. An aneurysm sac growing in contours after EVAR of minimal 10mm (per European  Guidelines4) as documented in the preceding 90 days by means of Echo Doppler (or  alternative visualization technique) and based upon sac diameter measurements; AND  6. An aneurysm sac that can be punctured via a translumbar approach; AND  7. Possibility to withhold anti-thrombogenic medication temporarily; AND  8. Ability and willingness to undergo the translumbar procedure under local anesthesia in a CT scanner; AND  9. Being older than 18 years. |
| --- |
| **Exclusion criteria:**  Patients are excluded from study participation if they meet ANY of the following criteria:  1. Patient not able or willing to give written Informed Consent; OR  2. Patient undergoing emergency procedures; OR  3. Patient with traumatic vascular injury; OR  4. Patients with an untreated open endoleak connected AMI (patients can be included if the AMI is coiled or is technically not possible to be coiled prior the ANEUFIX application);  5. Patient with hemostatic disorder (including bleeding disorders) or who is clinically  unstable; OR  6. Patient with a too high risk of abdominal sac rupture to allow safe radiological and  scanographic assessments; OR  7. Patient who is allergic to contrast media or anticoagulants; OR  8. Patient with renal impairment (eGFR < 30 ml/min); OR  9. Patient with a contra-indication for temporal positioning of a translumbar needle/catheter; OR  10. Patient who is participating in another trial with an investigational drug or medical device, or where a medical device/drug is used outside its labelling and its approved intended use; OR  11. Women of childbearing potential, OR  12. Patient with a life expectancy of less than 12 months. |
