# Supplementary material for: Initial Clinical Experience With AneuFix Injectable Biocompatible Elastomer for Translumbar Embolization of Type 2 Endoleaks
Source: J Endovasc Ther. 2023 Apr 19;32(1):57–67. doi: 10.1177/15266028231165731 (PMC11707960; doi:10.1177/15266028231165731)
Supplement: sj-docx-2-jet-10.1177_15266028231165731 – Supplemental material for Initial Clinical Experience With AneuFix Injectable Biocompatible Elastomer for Translumbar Embolization of Type 2 Endoleaks [file sj-docx-2-jet-10.1177_15266028231165731.docx]

## S2 Adverse events & definitions

This clinical investigation is executed in accordance with the ISO 14155 (2020) and MDR (EU) 2017/745. The definitions of AEs listed in the ISO-standard apply to the study.

All vascular related adverse events; ADEs, SAEs, SADEs, USADEs and serious health threats shall be

documented in a timely manner throughout the clinical investigation.

From the regulatory reportable device or vascular related AEs, sufficient information will be obtained

so as to permit 1) an adequate determination of the outcome of the event (i.e., whether the effect

should be classified as an SAE) and 2) an assessment of the causal relationship between the AE and

the investigational devices. The following information will be collected for those AEs that require

regulatory reporting:

• Title of event

• Start date of event

• Intensity of event

• Frequency

• Outcome

• Relationship to device/procedure

• Seriousness criteria

• Action taken

Instructions will be given to the local study team with respect to the contact person at the Sponsor in

case of observation of SAEs, SADEs and USADEs.

Vascular-related AEs occurring in any patient who signed the informed consent form will need to be

documented between the time the subject signed the informed consent and the time the subject

departs the study after the month 24 follow-up phone call. If a patient withdraws from the study prior to ANEUFIX treatment or is considered a screen failure, AEs until that study point need to be reported, but there are no further follow-up requirements. If the patient was enrolled in the study but withdrew consent after ANEUFIX treatment, reportable AE’s will be documented minimally during a 4-week follow-up period.

Safety data will be reviewed by the Data Safety Monitoring Board, the Coordinating Investigator and a representative of the sponsor to decide on complete documentation, device relatedness and impact on risk assessment. Recommendations to reduce the risk will be made and documented. Appropriate measures will be implemented by the sponsor as based on the recommendations received. During any time of the process, attention should be paid to signals that can indicate a serious health threat. Signals from adverse events or device deficiencies that might indicate a serious health threat can be detected by either the sponsor or principal investigator but are evaluated by the sponsor.

***Classification of Adverse Events***

| **Adverse events** | **Non-device related** | **Device or procedure related** | |
| --- | --- | --- | --- |
| Non-Serious | Adverse Event (AE) | Adverse Device Effect (ADE) | |
| Serious | Serious Adverse Event (SAE) | Anticipated | Unanticipated |
|  |  | Anticipated Serious | Unanticipated Serious |
|  |  | Adverse Device Effect (ASADE) | Adverse Device Effect (USADE) |

**Definitions:**

| **ADE** | Adverse event related to the use of an investigational medical device.  Note 1: This definition includes adverse events resulting from insufficient or inadequate instructions for use, deployment, implantation, installation or operation, or any malfunction of the investigational medical device.  Note 2: This definition includes any event resulting from use error or from  intentional misuse of the investigational medical device.  Note 3: This includes comparator if the comparator is a medical device. |
| --- | --- |
| **AE** | Untoward medical occurrence, unintended disease or injury, or untoward  clinical signs (including abnormal laboratory findings) in subjects, users or other persons, whether or not related to the investigational medical device and whether anticipated or unanticipated.  Note 1: This definition includes events related to the investigational device or the comparator.  Note 2: This definition includes events related to the procedures involved.  Note 3: For users or other persons this definition is restricted to events related to the investigational medical devices or comparators. |
| **Vascular-related AE** | An AE that relates qua origin or cause to the human vasculature or the blood. More specifically, these AEs are typically related to the AAA, the vascular wall, the EVAR procedure and post-EVAR complications (e.g. endoleak). |
| **SAE** | Adverse event that led to any of the following:  a) death  b) a serious deterioration in the health of the subject, users or other  persons as defined by one or more of the following:  - a life-threatening illness or injury, or  - a permanent impairment of a body structure or a body function  including chronic diseases, or  - in-patient or prolonged hospitalisation, or  - medical or surgical intervention to prevent life-threatening illness  or injury or permanent impairment to a body structure or a body  function  c) fetal distress, fetal death, a congenital abnormality, or birth defect  including physical or mental impairment  Note: Planned hospitalization for a pre-existing condition, or a procedure  required by the CIP, without serious deterioration in health, is not  considered a serious adverse event. |
| **SADE** | Serious Adverse Device Effect: ADE that has resulted in any of the  consequences characteristic of a SAE. |
| **USADE** | Unanticipated serious adverse device effect: SADE which by its nature,  incidence, severity or outcome has not been identified in the current version of the risk assessment. |
| **ASADE** | Anticipated serious adverse device effect: effect which by its nature, incidence, severity or outcome has been identified in the risk assessment. |
| **Device deficiency** | Inadequacy of the medical device with respect to its identity, quality, durability, reliability, usability, safety or performance.  Note1: Device deficiencies include malfunctions, use errors, and inadequacy in the information supplied by the manufacturer including labelling.  Note2: This definition includes device deficiencies related to the investigational medical device or the comparator |
| **Serious health threat** | Signal from any adverse event or device deficiency that indicates an imminent risk of death or a serious deterioration in the health in subjects, users or other persons, and that requires prompt remedial action for other subjects, users or other persons.  Note: This would include events that are of significant and unexpected nature such that they become alarming as a potential serious health hazard or possibility of multiple deaths occurring at short intervals. |
