# Supplementary material for: Initial Clinical Experience With AneuFix Injectable Biocompatible Elastomer for Translumbar Embolization of Type 2 Endoleaks
Source: J Endovasc Ther. 2023 Apr 19;32(1):57–67. doi: 10.1177/15266028231165731 (PMC11707960; doi:10.1177/15266028231165731)
Supplement: sj-docx-3-jet-10.1177_15266028231165731 – Supplemental material for Initial Clinical Experience With AneuFix Injectable Biocompatible Elastomer for Translumbar Embolization of Type 2 Endoleaks [file sj-docx-3-jet-10.1177_15266028231165731.docx]

## S3. Investigation procedures

|  | **Screening**  *Day 0-max90days* | | **ANEUFIX procedure** | | **Follow-up study period** | | | | | | |
| --- | --- | --- | --- | --- | --- | --- | --- | --- | --- | --- | --- |
| Assessments | **PRE-study** | **Eligibility / clinical data^1^** | **BASELINE**  *D0* | **Pre and end**  *D0* | **<24h/**  **Hospital**  **Discharge** | **1Week**  ± 6 days | **1** **Month**  ±10 days | **3 Months**  ±15 days | **6 Months**  ±30 days | **12 Months**  ±30 days | **24 Months**  ±30 days |
| Informed consent | X |  |  |  |  |  |  |  |  |  |  |
| Inclusion criteria | X |  |  |  |  |  |  |  |  |  |  |
| Exclusion criteria |  | X |  |  |  |  |  |  |  |  |  |
| Medical & EVAR/AAA history |  | X |  |  |  |  |  |  |  |  |  |
| Blood sampling: eGFR / sCR |  | X |  |  |  |  |  |  |  |  |  |
| Blood sampling: CRP |  |  | X |  |  |  |  |  |  |  |  |
| Blood sampling: CK |  |  | X |  |  |  |  |  |  |  |  |
| ANEUFIX procedure |  |  |  | X |  |  |  |  |  |  |  |
| Medication registration |  | X | X |  | X | X | X | X | X | X |  |
| Neurological examination |  |  | X |  | X | X^4^ | X^4^ |  |  |  |  |
| CTA scan |  | X |  | (X)^3^ | X |  |  | X | X | X |  |
| Ultrasound (optional) |  |  |  |  |  | X | X |  |  |  |  |
| Angiogram |  |  | X | X |  |  |  |  |  |  |  |
| AAA diameter measurements / endoleak presence/type^2^ |  | X |  | X | X |  |  | X | X | X |  |
| Adverse events |  |  | X | X | X | X | X | X | X | X | X |
| Patient status (telephone call) |  |  |  |  |  |  |  |  |  |  | X |

1 Patient data documentation can be of historic nature for medical history, CT scan and blood sample values (up to 90 days); other parameters should reflect current (max 30 days) status

2 At screening the (coded and preferably already existing) CTA scan is used and during the procedure the angiogram or CT scan (what is available) is used for the AAA diameter measurements and endoleak assessment. A CTA scan within 24 hrs of the procedure is required per protocol to document the outcome and to help assess the primary endpoint. The AAA sac growth is assessed using the CT scans of 3-6 and 12 months using

scans which are obtained through the standard follow-up procedure of EVAR patients.

3 If no CT scan (max 180 days old, but preferably <90 days old) prior to procedure is available, a CT scan can also be performed prior to the procedure (refer to inclusion criterium 4).

4 The neurological examination (standardized questionnaire) can be performed via a phone call with the patient, whereas during and after the ANEUFIX procedure, an independent physician will perform a standardized neurological
